# Pellagra: 4 D’s and 8 Points

**DOI:** 10.47795/FBFD9966

**Published:** 2020

**Authors:** Adrian Williams

**Affiliations:** Professor of Neurology in Birmingham, UK

## Abstract

Pellagra has largely been forgotten. This is unfortunate as important lessons are to be learnt for the diseases and social consequences of poverty (and of affluence) that often involve dietary nicotinamide and nicotinamide adenine dinucleotide (NAD) homeostasis. NAD disruption can occur not only from poor diet but from increased consumption of NAD from genotoxic and other stresses. High doses of nicotinamide lead to inhibition of NAD-consuming enzymes and excessive induction of nicotinamide-n-methyl transferase (NNMT) with consequent effects on the methylome giving a mechanism for a new hypervitaminosis-B3.

## Introduction

The history of Pellagra has been largely forgotten even if the 4 ‘D’s’ of Dementia, Dermatitis, Diarrhoea and Death are still taught to medical students.^1^, ^2^

Few realise that a festinating gait, fasciculation of the tongue or myoclonic encephalopathy were first described in the pellagra epidemics and other close mimics of many neurodegenerative and neuropsychiatric diseases were seen.

Pellagrins were prone to both dysbiotic and acute infections explaining the gut manifestations and the high incidence of TB. It was widely believed to be hereditary and certainly ran in families.

The original 18thC European epidemics affected poor peasants on monophagic maize based polenta diets. The early 20th C American epidemic predominantly affected poor blacks (and whites) often working as semi-slave sharecroppers thrown into poverty with the collapse of the cotton market and eating maize, molasses and small quantities of low quality pork.

Pellagra was believed to be degenerative in the dehumanising and atavistic sense of the term. Homo sapiens evolved on a high meat high nicotinamide diet so pellagra can now be seen as an example of human evolution in reverse. Inferior cognition and antisocial and addictive behaviours led to discrimination as the degenerate “Butterfly caste” contrasting with most societies that have ruling “meat elites”. Pellagrins were seen a different race having a different physiognomy that crossed colour lines even though it was an archetypal disease of poverty, rather like TB. Pellagrins were at times subject to and used as an exemplar to justify eugenic thinking.

Pellagra was a systems failure causing premature ageing with evidence of mitochondrial failure, oxidative stress and proteinopathy. Pellagra was curable particularly after the discovery of nicotinamide in the 1940s and is normally sourced from animal products as Vitamin B3. There were many undiagnosed and untreated cases as the exaggerated sunburn rash (Casal’s necklace) was often not present (“pellagra sine pellagra”) particularly in those with pigmented skin.

Point 1. A disorder that mimicked many neurodegenerative conditions as now classified can have a single and simple dietary cause even when there is evidence for (epi-) genetic involvement, dysbiotic microbiomes, mitochondrial and oxidative stress, and proteinopathy. Dietary nicotinamide with back-up from the degradation of tryptophan on the kynurenine pathway are the precursors to NAD critical to mitochondrial energetics as NADH (and other dehydrogenase reactions), anabolism as NADP(H), and NAD consumer pathways in an “NAD” world.^3^ Stress from toxins, whether chemical or microbial, requiring DNA or tissue repair by NAD consuming enzymes such as poly ADP ribose polymerases (PARPs) and Sirtuins could have the same pathological result if the dose of nicotinamide is not increased.

Point 2. Pellagra may be being missed. The better known clinical manifestations were recognised as being the “tip of the iceberg” in the epidemics in Europe and the south-eastern confederate states of the USA. Pellagra may be endemic in the millions in poverty who are meat and milk deprived masquerading as Kwashiorkor (“juvenile pellagra”) or “environmental enteropathy” or as poor cognition particularly in black populations who are resistant to the sunburn rash. A community screening test that would not be difficult to develop should be a priority.^4^

Point 3. Tuberculosis (TB), dysbiotic diarrhoeal illnesses and high death rates from acute infections, such as smallpox and measles, disappear as societies increase their meat and nicotinamide intake. This is no mystery as nicotinamide and its analogues, such as Isoniazid, are TB antibiotics and many bacterial toxins interact with NAD-consumer pathways – so being NAD replete would improve host resistance.^5^,^6^ Topically, COVID-19 interferes with the tryptophan uptake pathway and therefore NAD levels and T cell and Interferon responses through the Angiotensin-converting enzyme (ACE2) receptor through which it enters cells (that also malfunctions with mutations that lead to Hartnup disease that includes a pellagra-like syndrome). Some clinical manifestations, such as on gut and on cognition or long Covid, could be “formes fruste” or new versions of pellagra.^7^

Point 4. Reduction in many infections coincided with “meat transitions” and triggered demographic and epidemiological switches toward infertility and auto-immune, allergic and other diseases of modernity. Immune intolerance with changes in T cell subset ratios come about from less use of Indoleamine 2,3-dioxygenase (IDO) and the tryptophan to kynurenine “immune tolerance” pathway as in house production of nicotinamide is no longer necessary.^8^ Dietary modification of nicotinamide or tryptophan in diet, perhaps in concert with other vitamins such as Vitamin D, could affect the incidence of auto-immune conditions such as Multiple Sclerosis.^9^

Point 5. Meat transitions and “modernity” have, as if by magic, reduced the incidence of premature ageing, dementia, and death. Stunted lives were features of pellagra and in all species there are well described links between NAD metabolism and ageing, alongside resistance to infection, so no magic or much medicine is required. NAD levels fall with age and even further with many diseases of ageing and could respond to supplementation.^10^–^12^

Point 6. Early pellagra-ologists, such as Lombroso, may have been right in sensing that pellagrins were atavistic examples of degeneration in the 19th century sense of the term given that increasing meat intake was an important step in our evolution. For most of our evolution as hunter-gatherers we shared meat so the non-egalitarian meat variances that later developed are surprising and are now extreme with 100 fold variances across the globe between rich and poor. As a result, the poor particularly in poor countries face an adverse metabolic and transgenerational NAD headwind.^13^

Point 7. Inequalities of meat intake between classes and countries need to be fairer. Such extremes can be traced back to 17th century common pastureland “enclosure” movements and 19th century colonialism with the creation of the “third world” both channelling meat to the wealthy. Earlier extremes between the Old World and the New World that had few natural animal domesticates were corrected by the Columbian exchange enabling the rise of the West. However the global South was also unlucky in its meat supply, particularly Africa with a lack of animal domesticates and unhelpful human and veterinary infections in the tsetse fly belt with trypanosomiasis and rinderpest. Darker skin colour reduces the diagnostic help from sunburn and is a mixed blessing if it is acting as a warning of the more serious effects of nicotinamide deficiency compounding the problem. 4

Point 8. There may be a state of hypervitaminosis-B3. Many conditions, such as some cancers and metabolic syndromes common with affluence and a high meat intake (let alone nicotinamide supplementation) are linked to induction of the enzyme NNMT. NNMT detoxifies nicotinamide but consumes valuable methyl groups and nicotinamide overload might over-inhibit NAD-consumer enzymes that are metabolic master molecules.^14^–^16^ Nicotinamide’s methylated derivative resembles the dopaminergic neurotoxin MPTP and may, like nicotinamide, be a “double-edged sword”.

## Conclusion

Pellagra’s history is well worth remembering given that nobody systematically makes sure that it or “pellagra sine pellagra” is eliminated. Many in poverty remain at risk given that variances of meat intake are now historically extreme across countries and classes. Multi-organ pellagra was never “owned” by any one specialty but it should have remained a public health concern doubtless helped by supplementation but not helped by never being a universal policy. Nicotinamide’s potential toxicity in high meat economies was also never monitored over the long-term.

Dietary dosage or nicotinamide supplements may need to be boosted when individuals have certain mutations or are under stress, whether genotoxic or anoxic/metabolic – or restrained if there really is a hypervitaminosis B3 contributing to diseases of affluence. The environmental cost of optimising meat intake would be mitigated by affluent countries eating and wasting less but sharing more. The meat supply needs to be safe, with the poor not having to rely on “bush meat” or “wet markets” so as not to risk food poisoning or old and new zoonoses, such as COVID-19. Supplementation of nicotinamide alone may not be enough as animal products contain other helpful micronutrients such as iron and sources of methyl-groups such as choline and vitamin B12.

## Supplementary Material

Adrian Williams
