# Supplementary figures and images for: Pellagra: 4 D’s and 8 Points
Source: Adv Clin Neurosci Rehabil. Author manuscript; Available in PMC 2023 Dec 20. (PMC7615395; doi:10.47795/FBFD9966)

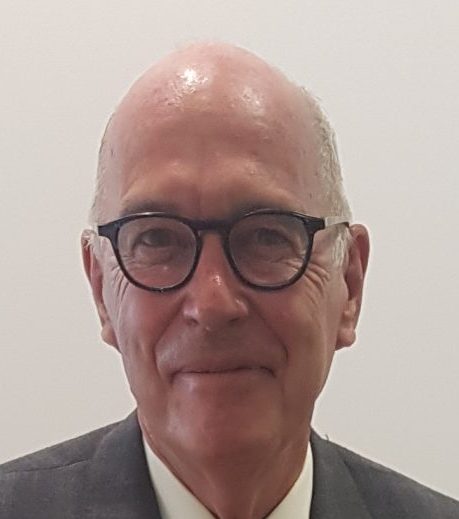

Supplement: Adrian Williams [file EMS103579-supplement-Adrian_Williams.jpg]
